# Family environment and prosocial behavior tendency of college students: The chain mediating role of empathy and moral sensitivity

**DOI:** 10.1371/journal.pone.0323375

**Published:** 2025-05-09

**Authors:** Huan Song, Shijie Fan, Yuan Zhao, Yue Wang

**Affiliations:** 1 School of Educational Science, Neijiang Normal University, Neijiang, China; 2 School of Psychology, Northwest Normal University, Lanzhou, China; 3 Police Officer Academy, Shandong University of Political Science and Law, Jinan, China; 4 Faculty of Education and Liberal Arts, INTI International University, Nilai, Malaysia; Alexandria University Faculty of Nursing, EGYPT

## Abstract

**Objectives:**

In order to explore the influence mechanism of family environment on the prosocial behavior tendency of college students and the chain mediating role of empathy and moral sensitivity in this relationship.

**Methods:**

A survey was conducted on 451 college students using the Family Adaptability and Cohesion scales, the Dispositional Moral Sensitivity Questionnaire, the Prosocial Tendencies Measure and the Chinese Version of the Interpersonal Reactivity Index-C.

**Results:**

The results showed that empathy and moral sensitivity significantly mediate the effect of family environment on college students’ prosocial behavior tendency. This is manifested in three mediation paths: the mediating role of empathy (with 28.57% of the mediating effect), the mediating role of moral sensitivity (with 61.90% of the mediating effect), and the chain mediating role of empathy and moral sensitivity (with 9.53% of the mediating effect).

**Conclusions:**

The study reveals the mechanism by which family environment affects college students’ prosocial behavior, which provides some theoretical guidance and practical inspiration for cultivating college students’ prosocial behavior.

## Introduction

The family environment has been demonstrated to play a significant role in the development of an individual’s prosocial behavior. Prosocial behavior tendencies are all behaviors and tendencies that people exhibit in social interactions, such as being accommodating, helpful, cooperative, sharing, and even sacrificing themselves for the benefit of others, that are conducive to social harmony [1,2]. In recent years, there has been an increasing number of studies on prosocial behavior tendency. Previous studies have shown that external social factors and internal individual factors are the two main aspects that affect prosocial behavior tendency. External social factors mainly include family environment [3,4], materialism [5], family function [6], etc. Intrinsic individual factors mainly include individual personality traits [7], emotional state [8], cognitive ability [9], etc. As one of the most important social environments in personal growth, family environment factors such as intimacy, adaptability, and parenting styles will all have an impact on an individual’s prosocial behavior tendency. Research shows that a negative family environment (e.g., parental conflict) is significantly negatively correlated with prosocial behavior, while a positive family environment (intimacy, adaptability) can positively predict prosocial behavior [3,4,10]. Therefore, the family environment plays an important role in shaping an individual’s prosocial behavior, and the internal connection and mechanism between the family environment and prosocial behavior tendency need to be further explored and discussed.

The family environment has been demonstrated to exert a significant influence on prosocial behavior tendencies, with these tendencies being expressed through heightened moral sensitivity. The tenets of social cognitive theory posit that environmental factors, individual cognitive processes, and individual behavior are inextricably linked. To illustrate, environmental factors (such as those within the family unit) can exert an influence on individual cognitive processes (such as empathy and moral discernment), which in turn can shape individual behavior (prosocial conduct) [11]. Among the numerous variables that may contribute to the influence of the family environment on prosocial behavior, moral sensitivity may serve as a mediator variable. Moral sensitivity can be defined as an individual’s capacity to discern the moral implications of a given situation and to respond to engage with moral issues in a nuanced and informed manner, drawing upon their genetic and moral experiences [12,13]. Empirical studies have demonstrated that the quality of the family environment can significantly and positively predict moral sensitivity [14], moral sensitivity is a significant and positive predictor of prosocial behavior tendencies [15,16]. In other words, individuals who demonstrate higher levels of moral sensitivity tend to exhibit more prosocial behavior. Guo posits that an individual’s cognitive schema exerts influence over their emotional responses and behaviors, as mentioned in the Cognitive Behavioral Theory (GBT) [17]. It is proposed that the family environment may enhance an individual’s moral sensitivity by shaping positive cognitive schemas, which in turn promote prosocial behavior. In light of the aforementioned evidence, this study proposes Hypothesis 1: moral sensitivity acts as a mediating variable between family environment and prosocial behavior tendency.

Moral sensitivity and empathy function as a mediating chain between the family environment and the tendency towards prosocial behavior. Empathy can be defined as an individual’s capacity to comprehend, empathise with and interpret the emotional states of others, albeit indirectly [18]. Empirical studies have demonstrated that an individual’s prosocial behavior is influenced by the family environment through the mechanism of empathy [3,19]. A favourable family environment, characterised by positive parenting styles and strong family relationships, is typically associated with secure attachment. A secure attachment relationship has been demonstrated to facilitate an individual’s emotional understanding and empathy for others, thereby enhancing prosocial behavior. Furthermore, empathy has been identified as an ethical emotion, with a substantial body of research indicating its role as a key driver and foundation for moral sensitivity development [14,20]. Individuals with higher levels of empathy are more likely to comprehend the circumstances in which others find themselves and to vicariously experience and empathise with their emotions. This facilitates an individual’s awareness of the moral content of a situation and their response to moral issues, which in turn results in higher moral sensitivity. In light of the aforementioned evidence, this study proposes Hypothesis 2: moral sensitivity and empathy serve as a mediating chain between family environment and prosocial behavior tendency.

In summary, this study built upon extant theories and research by investigating the relationship between family environment and prosocial behavior tendencies among college students. It also investigated the underlying mechanisms involved. The findings aim to provide a theoretical foundation for promoting prosocial behavioral development in university settings. The findings provide educators and parents with valuable insights and guidance for fostering pro-social behavior through empathy and moral sensitivity interventions.

## Methods

### Participants

In this study, Monte Carlo Power Analysis for Indirect Effects was utilized to calculate the sample size [21]. The following parameters were entered: Model = Two Serial Mediators, Power = 0.8, and Confidence Level = 99%. Additionally, the correlations and standard deviation between the variables were inputted. The results indicated that a sample size of 314 would achieve an effect size of 0.8.

A total of 474 questionnaires were returned, of which 451 were deemed valid, representing an effective response rate of 95%. The inclusion criteria for this study were as follows: full-time college students who voluntarily participated in the survey and completed it within 10–30 minutes. The exclusion criteria were as follows: surveys that took less than 10 minutes to complete, surveys that were incomplete, and surveys with patterned answers. The sample, aged 18–22 years, comprised 200 males (44.3%) and 251 females (55.7%). The sample was composed of 102 freshmen, 120 sophomores, 107 juniors and 122 seniors. The participants were given the option to take part in the questionnaire survey and were required to read and confirm the informed consent form prior to completing the questionnaire. The Research Ethics Committee of the School of Education Science at Neijiang Normal University (No. 2024–12) gave its ethical approval for this study, and all research procedures adhered to ethical guidelines.

### Procedure

Convenience sampling was used to recruit potential participants for this study, which was published online through the Questionnaire Star platform (https://www.wjx.cn/). Participants were drawn from a university in Neijiang, China, from February 25 to April 10, 2024. To obtain written informed consent from participants, an explanation of the purpose of the questionnaire was provided prior to the commencement of the survey, and participants were encouraged to respond with candor. An anonymous questionnaire was used and no personally identifiable information was collected.

Demographic information, including gender and grade level, was subsequently collected. Four scales were then completed by the participants: the Family Adaptability and Cohesion Scales, the Dispositional Moral Sensitivity Questionnaire, the Prosocial Tendencies Measure, and the Chinese Version of the Interpersonal Reactivity Index-C. At the conclusion of the survey, participants were thanked for their participation. It is important to note that participation was voluntary and anonymous, with no financial incentives offered for completion. All participants had the option to withdraw at any time.

### Measurements

#### Family adaptability and cohesion scales, FACES II.

The Family Adaptability and Cohesion Scales II, revised by Fei [22], was used. The scale consists of 30 items (‘When in trouble, family members will do their best to support each other’) and includes two dimensions: Cohesion (16 items) and Adaptability (14 items). Cohesion refers to the emotional connection between family members; adaptability refers to the ability of the family system to adapt to family situations and problems that arise at different stages of development. The original scale includes the actual feelings about the current situation of one’s family and the ideal family situation one would like to have. Only the actual feelings part of the scale was used in this study. A 5-point Likert scale was used, with 1 indicating ‘not at all consistent’ and 5 indicating ‘completely consistent’. A higher score indicates a higher degree of cohesion and adaptability, and a higher total score indicates a better family environment. This scale has been revised and proven to have good reliability [4]. In this study, the Cronbach’s α coefficient of this scale was 0.98, and the Cronbach’s α coefficients of each subscale were above 0.9.

#### Prosocial tendencies measure, PTM.

The Prosocial Tendencies Measure was initially developed by Carlo and revised by Kou [2]. It is a tool designed for the purpose of studying the prevalence of prosocial behavior. The scale comprises 26 items (‘When others ask me for help, I rarely refuse’), and includes six subscales: public, anonymous, altruistic, compliant, emotional and dire. A five-point Likert scale is employed, with 1 indicating ‘very unlike me’ and 5 indicating ‘very like me’. A higher score is indicative of a higher tendency to engage in prosocial behavior. The scale has demonstrated satisfactory reliability in samples of college students [9,23]. In this study, the Cronbach’s α coefficient of the scale was 0.976, and the Cronbach’s α coefficients for the subscales were all above 0.8.

#### Dispositional moral sensitivity questionnaire, DMSQ.

The Dispositional Moral Sensitivity Questionnaire, as devised by Zheng, was employed as a means of measuring moral sensitivity [24]. The questionnaire comprises 28 items (‘I will feel ashamed if I don’t uphold justice’), and is organized into five dimensions: empathetic guilt, punitive tendency, empathic annoyance, frequency of awareness, and empathetic imagination. A 6-point Likert scale was employed, with 0 indicating ‘strongly disagree’ and 5 indicating ‘strongly agree’. A higher score indicates a higher level of moral sensitivity. The scale has been shown to exhibit good reliability in samples of college students [25], and in this study, the Cronbach’s α coefficient for the scale was 0.98.

#### Interpersonal reactivity index-C, IRI-C.

The Interpersonal Reactivity Index-C (IRI-C) developed by Davis and revised by Zhang measures self-reported empathy of college students from a cognitive and affective perspective [26,27]. The scale comprises 22 items and includes four dimensions: Perspective Taking, Fantasy, Empathy Concern, and Personal Distress. The scale employs a 5-point scoring method, with 1 indicating ‘not at all consistent’ and 5 indicating ‘fully consistent’. A higher score indicates a greater capacity for empathy. The scale has been shown to exhibit good reliability in college student populations [28], and in this study, the Cronbach’s alpha coefficient for the scale was 0.96.

### Statistical analysis

The data were processed using SPSS 22.0 and Amos 24.0. A Harman single-factor test was employed to ascertain the presence of common method bias. During the subsequent phase of data analysis, unique codes were assigned to participants with the aim of preventing identification, and the principal investigator was the only person with access to the data. The results indicated that a total of 7 factors with eigenvalues greater than 1 were extracted, with the maximum factor variance explained being 35.30%, which is below the critical standard of 40%. Therefore, there is no significant common method bias in this study.

## Results

### Correlation analysis of variables

Descriptive statistics and correlation analysis were performed on the variables, and the results are presented in Table 1. The results of the correlation analysis indicated that, with the exception of gender, all variables exhibited a statistically significant positive correlation with one another (p < 0.001).

**Table 1:** Descriptive statistics and correlation analysis of family environment, prosocial behavior tendency, moral sensitivity and empathy.

Variable	M	SD	1	2	3	4	5	6	7	8	9	10
1 Cohesion	3.33	0.93	1									
2 Adaptability	3.32	0.93	0.95***	1								
3 Moral sensitivity	3.09	1.08	0.39***	0.40***	1							
4 Empathy	3.38	0.88	0.42***	0.44***	0.43***	1						
5 Public	3.39	0.90	0.44***	0.46***	0.50***	0.43***	1					
6 Anonymous	3.38	0.97	0.43***	0.46***	0.48***	0.42***	0.93***	1				
7 Altruistic	3.36	0.94	0.41***	0.43***	0.47***	0.41***	0.95***	0.85***	1			
8 Compliant	3.44	0.95	0.38***	0.39***	0.47***	0.39***	0.94***	0.85***	0.86***	1		
9 Emotional	3.36	0.95	0.42***	0.43***	0.50***	0.41***	0.95***	0.86***	0.89***	0.87***	1	
10 Dire	3.41	0.95	0.44***	0.44***	0.46***	0.40***	0.95***	0.86***	0.87***	0.88***	0.89***	1
11 Gender	–	–	-0.02	-0.02	0.05	0.03	-0.03	-0.01	-0.02	-0.06	-0.02	0.01

Gender is a dichotomous variable (1 = male, 0 = female); *indicates *p* < 0.05, **indicates *p* < 0.01, ***indicates *p* < 0.001.

### Chain mediation effect test of moral sensitivity and empathy

**Fig 1:**
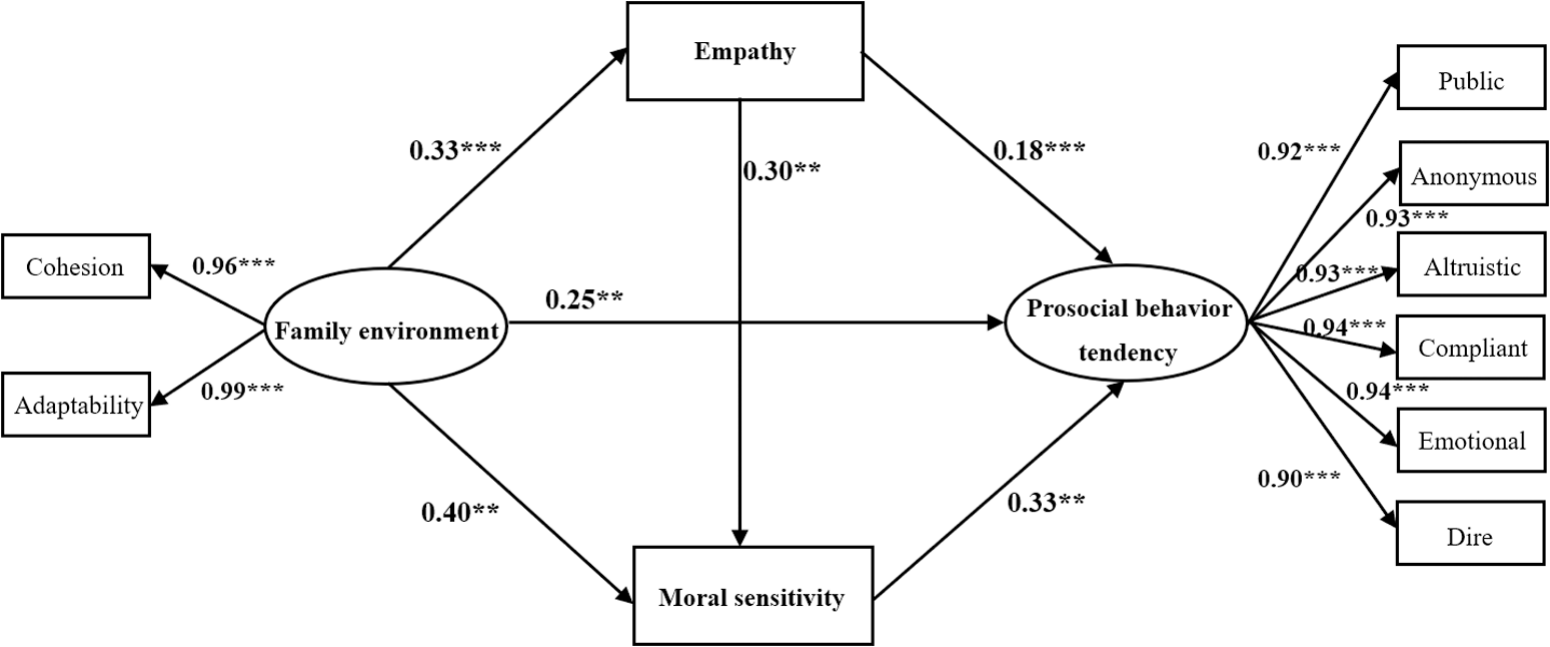
A chain-mediated model of moral sensitivity and empathy.

A structural model was established on the basis of the assumption that the model and the variables are correlated, with two dimensions of the family environment as independent variables, six dimensions of prosocial behavior tendency as dependent variables, and moral sensitivity and empathy as mediating variables (Fig 1). The Bootstrap method was used to test the mediating effect. The model exhibited an excellent fit (x^2^/*df* = 1.27, NFI = 0.992, GFI = 0.983, CFI = 0.998, IFI = 0.998, RMSEA = 0.024). As illustrated in Fig 1, the direct path from family environment to prosocial behavior tendency is significant (β = 0.25, *p* < 0.01), indicating that family environment can significantly and positively predict prosocial behavior tendency. Furthermore, family environment positively predicts moral sensitivity (β = 0.40, *p* < 0.01), and moral sensitivity positively predicts prosocial behavior (β = 0.33, *p* < 0.01), indicating that moral sensitivity plays a mediating role between family environment and prosocial behavior tendency. The family environment has a positive predictive effect on empathy (β = 0.33, *p* < 0.001), and empathy has a positive predictive effect on prosocial behavior tendency (β = 0. 18, *p* < 0.001). This indicates that empathy plays a mediating role between family environment and prosocial behavior tendency. Moral sensitivity positively predicts empathy (β = 0.30, *p* < 0.01), indicating that moral sensitivity and empathy play a chain mediating role between family environment and prosocial behavior tendency.

Meanwhile, the results of the Bootstrap mediation effect test further found that the 95% confidence interval of the total indirect effect of moral sensitivity and empathy on the family environment and prosocial behavior does not contain 0, indicating that their mediating effects are significant. As illustrated in Table 2, the mediating effect of moral sensitivity between family environment and prosocial behavior tendency is significant, with an effect size of 0.28. Moreover, the mediating effect of empathy between family environment and prosocial behavior tendency is also significant, with an effect size of 0.13. The chain mediating effect of moral sensitivity and empathy between family environment and prosocial behavior tendency is also significant, with a chain mediating effect of 0.04.

**Table 2:** Chain mediation effect of moral sensitivity and empathy and their effect sizes.

Path	Effect value	Effect size	Proportion of mediating effect	Bootstrap95%CI
Family environment→Empathy→prosocial behavior tendency	0.06	0.13	28.57%	[0.03,0.10]
Family environment→Moral sensitivity →prosocial behavior tendency	0.13	0.28	61.90%	[0.09,0.18]
Family environment→Empathy→Moral sensitivity→prosocial behavior tendency	0.02	0.04	9.53%	[0.01,0.04]
Total Mediation Effect	0.21	0.46		[0.16,0.34]
Direct effect	0.25	0.54		[0.16,0.27]
Total effect value	0.46			[0.39,0.53]

Effect size = Effect value/Total effect value; Proportion of mediating effect = Effect value/Total mediating effect.

## Discussion

This study, based on social cognitive theory, examined the internal mechanism by which empathy and moral sensitivity in the family environment influence prosocial behavior tendencies of college students. The findings showed that the influence of the family environment on prosocial behavior tendencies of college students is achieved through the chain mediation of empathy and moral sensitivity. In light of the aforementioned findings, the relationship between the family environment and college students’ prosocial behavior tendencies is subjected to detailed examination.

Firstly, this study found that the direct effect of family environment on prosocial behavior tendencies of college students is significant. A favourable family environment (the closer the relationships between family members, and the stronger the ability of family members to adapt to family situations and problems at different stages of family development) can directly enhance individuals’ prosocial behavioral tendencies. This result is consistent with previous research [3,4,6]. The family constitutes the most fundamental environmental factor in the growth process of college students, and it plays a pivotal role in the development of their prosocial behavior.

Empathy is generally considered to be a key factor influencing prosocial behavior. Previous studies have found that empathy can play a mediating role in the influence of the family environment on prosocial behavior [3,10]. This study provides further evidence to support this finding. This indicates that a positive family environment can facilitate college students’ empathy level, thereby enhancing their prosocial behavior tendencies. A favourable family environment is typically associated with a secure attachment. A secure attachment relationship has been demonstrated to facilitate the development of an individual’s empathy, which in turn enhances their propensity to engage in prosocial behavior [19]. A poor family environment is typically associated with negative attachment. For example, individuals with anxious or avoidant attachment may experience emotional indifference and emotional avoidance, which impedes the development of empathy and ultimately results in a reduced tendency to exhibit prosocial behavior [10].

Secondly, moral sensitivity also plays a mediating role in the process of family environment influencing prosocial behavior tendencies of college students, which verifies research hypothesis 1. Among the three mediating paths of family environment influencing college students’ prosocial behavior tendencies through empathy and moral sensitivity, the mediating effect of empathy was 0.13, the mediating effect of moral sensitivity was 0.28, and the chain mediating effect of empathy and moral sensitivity was 0.04. The mediating effect of moral sensitivity was the most significant, accounting for 61.90% of the total effect, which indicates that moral sensitivity is a crucial factor in the family environment’s influence on prosocial behavior tendency. The family is an important place for the formation of an individual’s moral sense. College students internalize and identify with the moral values conveyed in the family environment, which cultivates their moral sensitivity. This result has been confirmed by previous research, which found that family environmental quality can significantly and positively predict moral sensitivity [14]. Moral sensitivity, as an internal moral standard, can guide individuals in morally perceiving and evaluating social behaviors. Improving an individual’s moral sensitivity can help generate more prosocial behaviors [16]. Although there is currently no research directly demonstrating that moral sensitivity mediates the relationship between the two, according to socialization theory, an individual’s moral concepts are formed through interactions with family members and learning about social norms. In this process, the family environment provides models of moral norms and social behaviors. Consequently, individuals may gradually develop their own moral sensitivity through the imitation and internalization of social moral norms and behaviors. The ongoing evolution of one’s capacity to discern, comprehend, and interpret moral conduct in various scenarios will encourage individuals to engage in moral actions and deter immoral ones, thereby demonstrating prosocial behavior tendency. In particular, moral sensitivity was a stronger mediator than empathy, suggesting that it is a more direct and critical factor in influencing pro-social behaviour. The practical value of this finding lies in the need for schools and family education to pay more attention to cultivating students’ moral sensitivity. In terms of intervention program design, it is recommended to diverge from the traditional single model of empathy development and to design programs that focus on moral sensitivity.

In addition, the study revealed that the family environment has an impact on college students’ prosocial behavior tendencies through the chain mediation of empathy and moral sensitivity, which verifies research hypothesis 2. This shows that the family environment has an impact on an individual’s empathy and moral sensitivity, which in turn can have an impact on an individual’s prosocial behavior. Moreover, from the perspective of moral development, empathy is considered to be the foundation of moral reasoning and moral behavior. Empathy facilitates an individual’s understanding and response to the emotions of others, thereby enhancing an individual’s moral sensitivity. And, increased moral sensitivity improves an individual’s moral awareness, understanding, and interpretation of different situations, as well as their ability to identify and evaluate moral issues. This plays an important role in an individual’s prosocial behavior. As revealed by the chain mediation model, a positive family environment can facilitate college students’ empathy [10,14], and the improvement of empathy promotes the enhancement of moral sensitivity [19], which in turn promotes individuals’ tendency to exhibit more prosocial behaviors [16]. It can therefore be concluded that enhancing an individual’s empathy and moral sensitivity through improvements in the quality of the family environment is an important way to promote prosocial behavior.

### Conclusions

The mediating model constructed in this study not only confirms the conclusions of previous studies, but also further clarifies how family environmental quality affects prosocial behavior through empathy and moral sensitivity. The findings elucidate that family environment can indirectly influence college students’ prosocial behavior tendency through the chain mediating effects of empathy and moral sensitivity. The findings also elucidate the part played by empathy and moral sensitivity in this process, particularly the pivotal role of moral sensitivity. Furthermore, it offers a comprehensive examination of the mechanism through which family environmental quality, empathy and moral sensitivity influence prosocial behavior tendency, which has implications for enhancing college students’ prosocial behavior. It is imperative to consider the impact of the family environment on the prosocial behavior of college students. It is widely acknowledged that a positive family environment is conducive to the enhancement of an individual’s prosocial behavior. It is therefore essential to minimize or eliminate negative parenting styles and conflicts between parents, and to foster a positive family environment. Furthermore, family members should prioritize the cultivation of college students’ empathy and moral sensitivity, with the aim of promoting their prosocial behavior.

## Limitations and future directions

This study also has some limitations. Firstly, the cross-sectional study design employed in this study, which makes it difficult to verify the causal relationship between variables. It would be beneficial for future studies to employ a longitudinal tracking design in order to investigate the mediating mechanism of the family environment on prosocial behavior tendency, with a particular focus on chain mediation. Secondly, this study examines the mediating role of college students’ empathy and moral sensitivity in the relationship between family environment and prosocial behavior. Nevertheless, previous studies have indicated that moral identity may also be a significant mediating variable [6]. Therefore, future research could consider a greater number of variables in order to gain a deeper understanding of the internal mechanisms through which the family environment exerts an influence on prosocial behavior. In addition, the samples in this study were all from Chinese universities, making it difficult to generalize these findings to other groups. The measurement of family environment may be limited by the collectivist cultural context, whereas in individualistic cultures, the mechanism of family influence on pro-social behavior may be realized through different pathways. It is therefore recommended that future studies include a more diverse sample in order to increase the generalizability of the findings. Finally, socio-economic status (SES) was not taken into account in this study, and future research could further explore the influence of factors such as SES (e.g., parental education level, family income, etc.) on pro-social behavior.
